# Purple Sulfur Bacteria Dominate Microbial Community in Brazilian Limestone Cave

**DOI:** 10.3390/microorganisms7020029

**Published:** 2019-01-23

**Authors:** Eric L. S. Marques, João C. T. Dias, Eduardo Gross, Adriana B. de Cerqueira e Silva, Suzana R. de Moura, Rachel P. Rezende

**Affiliations:** 1 Department of Biological Sciences, State University of Santa Cruz, Rod. Jorge Amado, Km 16, Ilheus CEP: 45662-900, Bahia, Brazil; jctdias@gmail.com (J.C.T.D.); adrianabarros_ufba@hotmail.com (A.B.d.C.eS.); suzana.moura@yahoo.com.br (S.R.d.M.); 2 Department of Agricultural and Environmental Science, State University of Santa Cruz, Rod. Jorge Amado, Km 16, Ilheus CEP: 45662-900, Bahia, Brazil; egross@uesc.br

**Keywords:** Chromatiales, amoA, nifH, AOA, AOB, qPCR, Illumina, 16S rDNA

## Abstract

The mineralogical composition of caves makes the environment ideal for inhabitation by microbes. However, the bacterial diversity in the cave ecosystem remains largely unexplored. In this paper, we described the bacterial community in an oxic chamber of the Sopradeira cave, an iron-rich limestone cave, in the semiarid region of Northeast Brazil. The microbial population in the cave samples was studied by 16S rDNA next-generation sequencing. A type of purple sulfur bacteria (PSB), Chromatiales, was found to be the most abundant in the sediment (57%), gravel-like (73%), and rock samples (96%). The predominant PSB detected were Ectothiorhodospiraceae, Chromatiaceae, and Woeseiaceae. We identified the PSB in a permanently aphotic zone, with no sulfur detected by energy-dispersive X-ray (EDX) spectroscopy. The absence of light prompted us to investigate for possible nitrogen fixing (*nifH*) and ammonia oxidizing (*amoA*) genes in the microbial samples. The *nifH* gene was found to be present in higher copy numbers than the bacterial-*amoA* and archaeal-*amoA* genes, and archaeal-*amoA* dominated the ammonia-oxidizing community. Although PSB dominated the bacterial community in the samples and may be related to both nitrogen-fixing and ammonia oxidizing bacteria, nitrogen-fixing associated gene was the most detected in those samples, especially in the rock. The present work demonstrates that this cave is an interesting hotspot for the study of ammonia-oxidizing archaea and aphotic PSB.

## 1. Introduction

Caves offer a unique environment for microbial life, which is an aphotic, non-photosynthetic, and commonly oligotrophic environment, isolated to some degree from the surface. They present a distinct mineralogical composition, associated with rocks [1,2,3] that provide an opportunity for colonization by different microbial communities. Recently, there was an increase in the number of studies conducted on microbial communities in tropical caves, especially those close to the Equator line (such as Brazilian caves) [4,5,6,7,8]. However, these caves represent only a small fraction of the many existing tropical caves, and most of these studies are based on cultured fungi and bacteria [5,7]. Additionally, culture-based studies do not reveal complete information regarding the microbial diversity of a region. On the contrary, culture-independent techniques not only provide more detailed information regarding the microbial communities inhabiting the caves, but also reveal the various diverse and unexpected [6] metabolic activities that exist within the cave ecosystem [4,9,10].

Among the unexpected microbes found in caves are the Chromatiales, also known as purple sulfur bacteria (PSB), described in microbiology books as phototrophic anoxygenic bacteria that use hydrogen sulfide as an electron donor [11]. However, PSB, such as the ones belonging to the genus *Halothiobacillus,* grow in aphotic and aerobic conditions [12]. Despite their photosynthetic and sulfur metabolizing ability, PSB are commonly found associated with nitrogen fixation [13] and ammonia oxidation [14], carbon fixation and chemolithoautotrophy [15], and potentially perform these activities in the cave ecosystem. Previous studies showed PSB to be the dominant bacteria in photic zones of caves [16] and nondominant members of the microbial community in the aphotic zones, such as the yellow microbial community [17].

In this study, we focused on the bacterial community in Sopradeira cave, a limestone cave in the semiarid Northeast region of Brazil. There are few microbiological studies based in caves in the NE region of Brazil [4,5,18,19], which comprise an area of 1.55 million km^2^. Additionally, most of these studies were conducted by our group [4,18,19]. Among the 200 known caves of the karstic area of Sopradeira cave, only one microbiological study was conducted, which was a culture-based study on cellulolytic fungi in *Gruta do Catão* [5]. Therefore, the microbial diversity within these caves remains largely unexplored. The Sopradeira cave is at least 4 km long and is not open for tourism. It is located in the Neoproterozoic metamorphic limestones, outcropping from arenitic deposits. The cave’s rock presented a reddish color, uncommon in the area, and was found to be an iron-rich limestone cave. Therefore, we decided to analyze the microbial community in two types of samples (sediment and rock) by next-generation sequencing of 16S rDNA. We initially aimed to compare the composition of the bacterial community in oxic sediment and rock samples of Sopradeira cave. However, the predominant presence of Chromatiales in the aphotic environment led us to evaluate the hypothesis that these bacteria are associated with the nitrogen cycle, especially nitrogen fixation and ammonia oxidation.

## 2. Materials and Methods

### 2.1. Sampling and DNA Extraction

We collected approximately 100 g of sediment and gravel-like samples and a piece of approximately 100 g of rock sample from the same chamber of a cave called Buraco da Sopradeira (Sopradeira cave, 12°26′55″S 44°57′56″W) located in São Desidério, Bahia state, Brazil. The amount and number of samples were authorized by SISBIO/IBAMA/MMA No. 38453 and limited for protection of the site. The cave was selected based on its reddish color and size, which were essential factors for selection, among the 200 known caves in the area. The vertical projection of the cave and abyss makes this cave exclusively accessible to the scientific and speleological communities and the absence of visible drippings makes this cave a sparsely external-contaminated site. Therefore, the cave is suitable for analysis of the microbial community. We collected a composite sample of five subsamples of sediment and gravel-like samples (Figure S1 shows examples of sediment and gravel-like samples) 2 m away from each other in a 10 m^2^ area of the chamber. Sediment and gravel-like samples were collected from the surface to a depth of 2 cm (maximum depth of sediments). A rock sample was collected at a height of 1 m from the cave floor, located approximately 1 km from the entrance and stored in sterile plastic bags and kept on ice, until reaching the laboratory. DNA extraction was carried out using the MoBio PowerSoil DNA isolation kit (MoBio Laboratories, Carlsbad, CA, USA), following the manufacturer’s instructions. The rock sample was crushed using sterile mortar and pestles, prior to extraction. DNA extraction and preparation for the procedure, were performed in a laminar flow hood.

### 2.2. EDX, pH, Organic Matter, and Nitrogen Analysis

An EDX analysis was performed by scanning electron microscopy (model Quanta 250 (FEI Company). Stubs were used to mount each sample separately and three points were analyzed at 15 kV. Spectrographs are shown in Figure S2. At the laboratory, a part of the gravel-like and rock samples was crushed. Thereafter we sieved these and the sediment samples individually through a 2 mm mesh. Samples were sent to a service provider at the Soil Department of the Federal University of Viçosa for nitrogen analysis. Furthermore, analysis of organic matter, nitrogen, nitrate, and ammonia were performed following procedures developed by the service provider [20]. Measurement of pH was done following the protocol of Ribeiro et al. [20]. Samples were kept frozen until analysis.

### 2.3. 16S rDNA Sequencing

Amplification of the 16S rRNA V3/V4 region was carried out using the 341F (5′- CCT ACG GGR SGC AGC AG) and 806R (5′- GGA CTA CHV GGG TWT CTA AT-3′) primers [21,22]. The 16S rRNA libraries were constructed following PCR-based protocol [23] and sequenced using the MiSeq Sequencing System (Illumina Inc., San Diego, CA, USA) with the V2 kit, 300 Cycles. Reads were separated by barcoding and trimmed using Geneious 11.0.4. Chimeras were detected using decipher [24]. Operational taxonomic units (OTUs) were assigned by clustering the sequence against RDP database (version 2.12, https://rdp.cme.msu.edu/) [25] and EZBioCloud database (version 2017.05, https://www.ezbiocloud.net/) [26] using a cutoff of 97%, followed by the calculation of Shannon index and Good’s coverage index.

### 2.4. Quantitative PCR

Quantitative PCR (qPCR) was performed on the ABI prism 7500 fast system (Applied Biosystems, Foster City, CA, USA) to quantify *nifH*, archaeal *amoA*, and bacterial *amoA* genes in the samples. Amplification was performed with 1X of Kapa Sybr Fast qPCR master mix with ROX (Kapa Biosystems, Wilmington, MA, USA), 0.2 µM of each primer, and 2 µL of extracted DNA in a final volume of 20 µL. A standard curve was plotted with 10-fold dilutions of a cloned PCR product for each gene analyzed. All analyses were performed in triplicate. The amplification of *nifH*, archaeal amoA, and bacterial amoA genes were performed using the following primers: PolF (5′-TGC GAY CCS AAR GCB GAC TC-3′) and PolR (5′-ATS GCC ATC ATY TCR CCG GA-3′) for *nifH* [27]; Arch-amoAF (5′-STA ATG GTC TGG CTT AGA CG-3′) and Arch-amoAR (5′-GCG GCC ATC CAT CTG TAT GT-3′) for archaeal *amoA* [27]; and amoA1F (5′-GGG GTT TCT ACT GGT GGT-3′); and amoA2R (5′-CCC CTC KGS AAA GCC TTC TTC-3′) for bacterial *amoA* [27].

## 3. Results

### 3.1. 16S rDNA Sequencing

The 16S rDNA sequencing on Illumina platform resulted in at least 86,000 reads per sample. After processing, a minimum of 69,000 high-quality reads were obtained (Table 1). The gravel-like sample presented the highest numbers of OTU and highest diversity according to the Shannon index (Table 1). The rock sample presented the lowest number of OTU and an intermediate diversity. However, the Good’s coverage indicates that the rock sample is close to a complete estimate sampling of species/OTU in comparison with the other samples (Table 1). Similar results were found in rarefaction curves (Figure S3).

**Figure 1:**
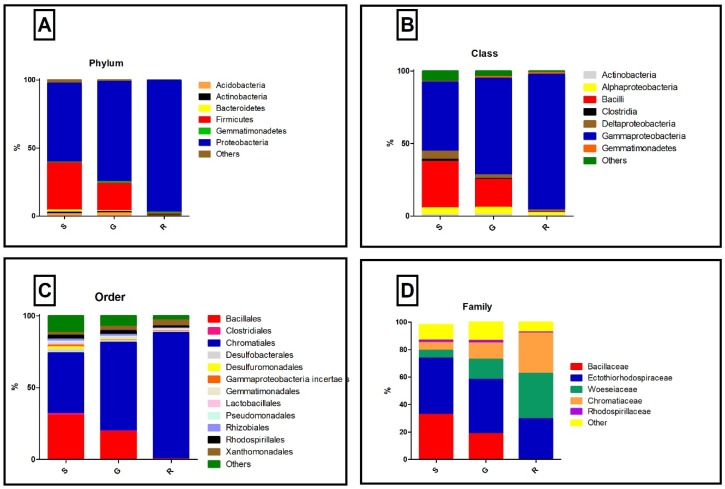
Taxonomic classification of 16S rDNA reads for the cave samples. Sediment (S), Gravel-like (G), and Rock (R) samples. (**A**) Phylum level, (**B**) Class level, (**C**) Order level, and (**D**) Family level with EzBiocloud database.

We detected a total of 17 phyla in the samples. Proteobacteria predominated in all samples with 57%, 73%, and 96% in samples S (sediment), G (Gravel-like), and R (rock), respectively, followed by Firmicutes (34%, 20% and 0.6%) and other phyla (Figure 1A). The predominant class in S, G, and R samples were Gammaproteobacteria (47%, 66%, and 93%, respectively), Deltaproteobacteria (5%, 2%, and 1%, respectively), Alphaproteobacteria (4%, 4%, and 2%, respectively) from the phylum Proteobacteria, and Bacilli (32%, 19%, and 0.3%, respectively) from the phylum Firmicutes (Figure 1B). Gammaproteobacterial order, Chromatiales, predominated in all three samples, S, G, and R with 47, 62, and 88%, respectively (Figure 1C), followed by Bacillales (30%, 19%, and 0.18% in samples S, G, and R, respectively). In the family analysis, we identified three families among the Chormotiales order: Ectothiorhodospiraceae, Chromatiaceae, and Woeseiaceae. However, Bacillaceae represented a major part of the microbial community in the S and G samples (Figure 1D). Ectothiorhodospiraceae was the most abundant family found in these samples, while in the rock sample, Chromatiaceae and Woeseiaceae presented a similar representation (Figure 1D).

We attempted to classify the microbial population up to the genus level by comparing our results with information available on two databases. However, the dominant genus remained unclassified, and were represented as ‘family_uc’ (for example, Chromatiaceae_uc), or the level of confidence for a specific genus was too low, including a small difference among the purple sulfur bacteria family’s distribution. The unclassified genera represented 80% of the rock sample and at least 50% of the other samples (Table S1).

It is noteworthy that concordance between results was observed when bacterial sequences were compared with both databases independently. Therefore, sequence comparison with each database resulted in identification of the same taxa, enabling accurate classification of these bacteria up to family and genus level. However, in RDP database version 2.12, we were unable to detect Woeseiaceae, as this newly described family was not present in the database. The genus *Nitrococcus*, a nitrifying Ectothiorhodospiraceae, was detected in rock, sediment, and gravel-like samples at 8.8%, 4.1%, and 6.6%, respectively. Moreover, *Bacillus* represented 35% (S), 19% (G), and 0.08% (R) of the bacterial community in those samples. *Steroidobacter*, a Gammaproteobacteria, was also found to be present in the samples, especially rock (1.5%).

The dominance of PSB in the absence of light and sulfur led us to analyze another important metabolic function of this group: Nitrogen metabolism. However, most of the dominant species were assigned with low confidence level and associated with nitrogen metabolism, such as *Nitrosococcus* as the dominant Chromatiales. This encouraged us to analyze nitrogen fixation and ammonia oxidation in the samples. Furthermore, a *nifH*-DGGE analysis (Figure S4) showed the presence of OTUs associated with two PSB: *Ectothiorhodospirceae* and *Chromatiaceae* (Text S1).

### 3.2. Quantitative PCR (qPCR)

**Figure 2:**
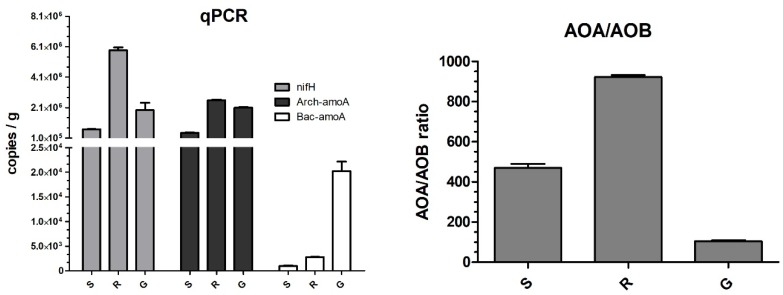
Quantitative PCR (qPCR) of genes associated with nitrogen fixation and ammonia oxidation and the archaeal and bacterial ammonia oxidation ratio (AOA/AOB). The bacterial nitrogen fixing gene (nifH), archaeal (AOA), and bacterial (AOB) ammonia-oxidizing genes in sediment (S), gravel-like (G), and rock sample (R). Assays were performed in triplicate (*n* = 3), bars represent standard deviation.

The frequency of *nifH* was found to be higher than bacterial-*amoA* by at least 100-fold (Figure 2). Moreover, qPCR analysis revealed that the frequency of *nifH* was the highest in the rock samples and lowest in the sediment samples. The gravel-like sample was found to have at least 10 times more copies of bacterial-*amoA* (AOB), as compared to the rock and sediment samples (Figure 2). Due to the low frequency of AOB in R and S samples, we decided to investigate the archaeal-*amoA* (AOA) in those samples (Figure 2). Arch-*amoA* was detected at 10^5^ to 10^6^ copies per gram, indicating the occurrence of ammonia oxidation in those samples. Almost 10-times more copies of the Arch-*amoA* was detected in the sediment samples, as compared to the rock and gravel-like samples. It is noteworthy that the *nifH* gene was detected at 10^5^ to 10^6^-fold in comparison with AOB, which was detected at 10^3^ to 10^4^-fold copies of the gene per gram.

The AOA/AOB ratio showed a dominance of AOA over AOB (Figure 2), at approximately 100-, 500- and 900-times in G, S, and R samples, respectively, indicating a considerable difference among the samples.

### 3.3. EDX and Nitrogen Analysis

We performed an EDX analysis in order to identify traces of elements in our samples. Among the identified elements described in Table 2, sulfur was detected in only one of the samples, while iron was a predominant component in all samples. The presence of iron ranged from 5% (sediment) to 17% (rock), explaining the reddish color of the samples. Silicon was present at nearly 15% in all samples, indicating a layer of arenite in the limestone rock. A small fraction of rock and gravel-like samples was dissolved with hydrochloride acid, following the observation of some resistant grains in the sediment sample after dissolution, probably due to the presence of silicon dioxide.

The rock sample was found to have higher content of ammonia and nitrate, while the sediment sample showed higher nitrogen content (Table 3). Ammonia, nitrate, and total nitrogen ranged from 42 to 75 mg/kg, 38 to 59 mg/kg, and 2.60 to 2.87 g/kg, respectively. The organic matter content was also evaluated. However, the results obtained were below the minimum detectable value (5 g/L) in all samples. The rock sample was found to have higher pH as compared to the other samples, which were close to neutral (pH 7), with the gravel-like sample being slightly alkaline and the sediment sample being slightly acidic (Table 3).

## 4. Discussion

The Brazilian caves, especially those in the Northeast located in the tropical zone [4,5,6], are scarcely explored in terms of their microbial diversity, with only a few existing microbiological studies to shed light on the microbial inhabitants in that region. We decided to work in the São Desidério area because the region is a biodiversity hotspot [28] inhabited by unknown microbial communities. The *Buraco da Sopradeira* was especially interesting to study due to the distinct reddish color of its rocks and different sediment granulometry (Figure S1). Predominance of the Chromatiales (also known as purple sulfur bacteria), detected in oxic and aphotic sections of the cave, was initially unexpected due to the popular association of this PSB with photosynthesis and anaerobic environments [11], and by the non-detection of sulfur in the EDX analysis. Moreover, previous studies in caves showed that the photosynthetic PSB are the dominant bacteria in photic zones of caves [16], rather than the aphotic zone, as detected in our study. Moreover, one of the dominant PSB identified in this cave chamber was a member of a relatively new family of facultative anaerobic PSB, Woeceiaceae [29], that was not included in one of the database used. However, this brings forth an important question: How do such bacteria enter the cave ecosystem and dominate the bacterial community?

It may be speculated that microorganisms, such as the PSB, entered the cave ecosystem many years prior. Considering that most of the limestone caves in Northeastern Brazil were either associated with flooding and/ or with a river during ancient times [30], it is plausible that a transport of microorganisms, such as PSB, including photosynthetic ones, (as previously detected, Cyanobacteria), took place near the cave river area [4]. Moreover, the indication of runoff, airflow, or a flowing river, albeit for a short duration since cave formation, can explain the higher presence and accumulation of Bacillaceae in the floor, as they were detected in greater abundance in the sediment samples. Interestingly, these Bacilli were found to increase with a decrease in pH, indicating that they might demonstrate some unknown metabolic activities. Moreover, the alkaline pH and the total absence of light in the sampling site supports the idea of a predominance of non-photosynthetic PSB. However, the failure to isolate PSB and the notion that photosynthetic activity is an ancient feature in PSB [31] encouraged us to further investigate whether the PSB in the Sopradeira cave could perform photosynthesis, had lost the genes needed for photosynthesis, or produced inactive photosynthetic-related proteins. As we did not expect the dominance of PSB, we were unable to try techniques to isolate them, including an anaerobic collection. Moreover, due to the restrictions in sampling, only one chamber was analyzed and the result may not be representative to the whole Sopradeira cave environment. Furthermore, the reduced sampling (approximately 100 g) that we were authorized restrict the geochemical analysis feasible to be performed. The lack of geochemical information restricts our understanding on PSB metabolism and how it thrives in cave ecosystem. 

Among the possible activities that PSB may be related to in the cave are carbon fixation [16] and sulfur metabolism. However, the absence of light and detectable sulfur in the sampling site decreased the possibility of these two metabolic activities performed by the PSB. Nonetheless, non-photosynthetic carbon fixation may not be altogether disregarded. Nitrogen metabolism is known for more than 60 years in PSB [32], and a recent study showed the presence of the *nifH* gene in several members of Chromatiales [13]. This corroborates our finding by qPCR and 16S rDNA sequencing that *nifH* was present at a high frequency. The high-intensity bands observed on *nifH-*DGGE analysis (Figure S4) made with the same volume of PCR product, confirming the presence of the *nifH* in high copy numbers in the rock sample. Indeed, the level of potential nitrogen fixating bacteria observed in the present study was 100-times higher than a previous study from our group in another limestone cave [4] and supported the idea of PSB related with nitrogenase activity in the studied chamber of Sopradeira cave. The surprisingly high level of *nifH* was unexpected because of the amount of energy spent to fix one N_2_ molecule [33]. However, it is plausible to assume that part of the *nifH* detected in the samples may not form an active nitrogenase, since the presence of *nifH* does not guarantee expression and full function of this multigenic enzyme [34]. However, it is a strong indication regarding a potential activity, albeit the nitrogenase enzyme is known to be involved in other reactions [35]. Furthermore, the presence of *nifH* gene does not guarantee nitrogen fixing activity, and these bacteria may have other genes related with biogeochemical cycles, indicating that despite the presence of nitrogenase, these bacteria may also be associated with other biochemical reactions. 

Curiously, the total levels of nitrogen, ammonia, and nitrate determined in our study were similar to our findings from a previous study in another cave, where 100-times less *nifH* copies per gram were detected [4], which may be a relatively constant number for tropical caves. It is interesting to note that the *nifH* and ammonia (nitrogen fixation product) values were higher in the sample with more PSB abundancy (rock) and higher pH. The rock sample in DGGE (Figure S4) presented a high number of bands in spite of using different primers for *nifH* amplification, and the sequenced bands matched with Chromatiales in the NCBI database (Text S1). These findings, the Good’s coverage value, rarefaction curve (Figure S3), and the abundance of bacteria support our assumption that PSB in Sopradeira cave is associated with nitrogenase enzyme, especially considering the fact that no other bacterial group detected in the study had the potential to present such high copy numbers of the *nifH* gene. It is interesting to note that, in another iron-rich cave sample (from ferromanganese deposit) [10], nitrogencycle-related bacteria and Bacilli from different families were detected. In the present study, the microorganisms transported to the cave appear to have adapted to perform a similar or the same function.

Initially, we envisaged that PSB in the cave samples were ammonia-oxidizers. However, the difference between AOB and *nifH*, evident from our qPCR results, suggests a greater presence of potential nitrogen-fixing bacteria than AOB in the samples, in addition to the presence of multiple copies of these genes per genome [36]. The predominance of PSB inside the cave may be attributed to the *nifH* gene due the high copy numbers. However, a small fraction of the AOB present, appeared to be PSB, as identified by 16S rDNA sequencing, albeit by low-confidence identification. These bacteria were identified as *Nitrosococcus*, a known ammonia-oxidizer [36]. The observation that AOB were only a small percentage of the total microbial population inhabiting the cave prompted us to investigate if AOA were the dominant ammonia -oxidizing bacteria in the samples. Interestingly, AOA was found to be approximately 10- (Sediment) to 100-times (rock and gravel-like) more as compared to the findings of our previous study in another Brazilian cave [4]. Thereafter, the AOB population seemed to present the same level leading to an AOA/AOB ratio, which was higher than that observed in the other study, especially considering that AOB genomes have one to three copies of the *amoA* gene, while known the archaeal genome only has one copy [37].

## 5. Conclusions

The predominance of PSB brings many questions, but the limited funding, especially during the current Brazilian science crisis [38], narrowed our possibilities to better characterize the environment and investigate a broad of potential PSB activities in caves and remnants of their life in surface. The predominance of PSB observed in the present study is one of many examples of diverse and unexpected microbial colonization [6,8,10], making the caves an exciting place to study unique microbial communities. Nevertheless, in most countries, the legislation neglects microbial communities in caves. The Brazilian legislation, for example, classified caves according to a rank scheme of protection mainly related with geology and zoology. A recent study proposed alternative methods [39], but failed to mention microorganisms. NGS techniques became affordable in recent years, enabling its application in diversity analysis and facilitating the inclusion of evaluating the microbial community in cave (and many other) legislation around the world. This will not only encourage the study of unique microbial communities, like the one in the present study, but also open potential avenues for discovery of new biotechnologically interesting biomolecules [40] that represent an important genetic patrimony. To the best of our knowledge, our group is the first to demonstrate the predominance of purple sulfur bacteria in dark zones of cave samples. Our study suggests that PSB in the Sopradeira cave participate in the nitrogen cycle. The study also improves the knowledge of PSB in oxic, aphotic, and non-sulfurous environments. However, more studies need to be conducted to investigate the PSB activity in such caves. Due to the unexpected predominance of these bacteria and our focus on the biotechnological potential in the bacterial isolation technique, we were unable to isolate PSB from our samples. However, future work may be directed towards the isolation of such bacteria from this cave and investigate if PSB in aphotic zones still have photosynthetic potential. Nevertheless, the analyzed chamber in the Sopradeira cave presents an opportunity for studying ammonia-oxidizing archaea and a diverse microbial community of PSB in the aphotic environment to improve the understanding of the Chromatiales class in different ecosystems.

## Figures and Tables

**Table 1:** Summary of 16S rDNA sequencing and analysis of Sopradeira cave samples. R (rock), G (gravel-like), and S (sediment).

Samples	Raw Reads	Reads after Processing	OTU	Shannon	Good’s Coverage (%)
**R**	86,230	69,751	2640	5.3	99.94
**G**	176,263	91,131	3802	5.7	99.86
**S**	125,347	72,822	2667	4.7	99.89

**Table 2:** Energy-dispersive X-ray spectroscopy analysis of cave sediment (S), gravel-like (G), and rock (R) samples. Values in % followed by the standard deviation.

Samples	C	O	Fe	Mg	Al	Si	K	Ca	S
**S**	5.04 ± 4.56	62.95 ± 4.84	4.96 ± 1.54	0.00	11.52 ± 2.8	14.98 ± 4.59	0.00	0.54 ± 0.42	0.00
**G**	11.55	51.28 ± 1.41	12.40 ± 1.82	0.66 ± 0.42	8.56	14.62 ± 4.31	0.07 ± 0.06	0.43 ± 0.29	0.44 ± 0.35
**R**	6.78 ± 2.16	50.471.82	16.91 ± 1.88	0.00	9.24 ± 3.4	15.90 ± 2.91	0.00	0.71 ± 0.14	0.00

**Table 3:** Total nitrogen, ammonia, nitrate (Values in g/kg), and pH of cave sediment (S), gravel-like (G), and rock (R) samples.

Samples	Nitrogen	Ammonia	Nitrate	pH
**S**	2.87	0.042	0.046	6.4
**R**	2.83	0.075	0.059	8.9
**G**	2.60	0.067	0.038	7.6

## Supplementary Materials

The following are available online at http://www.mdpi.com/2076-2607/7/2/29/s1, Figure S1: Images of the size of sediment (A), gravel-like (B) samples, and a piece of the rock sample; Figure S2: Spectrographs generated in the EDX analysis of Sopradeira cave samples; Figure S3: Rarefaction curves of rock, sediment and gravel-like samples from Sopradeira cave; Figure S4: Denaturing Gradient Gel electrophoresis (DGGE) of nifH gene community in the rock (R), sediment (S), and gravel-like (G) samples; Table S1: List of genus and family identify in the 16S rDNA sequencing of sediment (S), gravel-like (G), and rock (R) samples collected in Sopradeira cave. Text S1: Denaturing Gel Gradient Electrophoresis of *nifH* gene.
