# Supplementary material for: Purple Sulfur Bacteria Dominate Microbial Community in Brazilian Limestone Cave
Source: Microorganisms. 2019 Jan 23;7(2):29. doi: 10.3390/microorganisms7020029 (PMC6406701; doi:10.3390/microorganisms7020029)
Supplement: Supplementary file 1 [file microorganisms-07-00029-s001.zip › microorganisms-408874-supplementary materials.pdf]

Purple Sulfur Bacteria Dominate Microbial Community in Brazilian Limestone Cave

Eric L. S. Marques *, João C. T. Dias, Eduardo Gross, Adriana B. de Cerqueira e Silva, Suzana R. de Moura, and Rachel P. Rezende *

Text S1. Denaturing Gel Gradient Electrophoresis of *nifH* gene.

The Denaturing Gel gradient electrophoresis (DGGE), band excision and sequencing procedure were performed according Marques et al. [4].

The sequenced bands were identified as *Ectothiorhodospira* (EF199955, 3 bands with 97, 98 and 98% of similarity), *Thiocapsa* (EU622783, one band with 98% of similarity), *Thioalkalispira* (CP001905, one band with 99% of similarity) as the closest match in GenBank database.

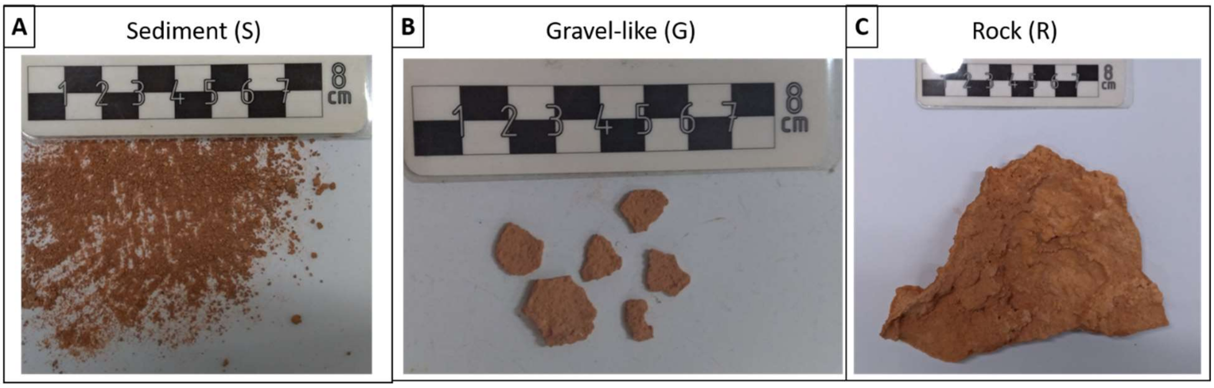

Figure 1. Images of the size of sediment (A), gravel-like (B) samples and a piece of the rock sample.

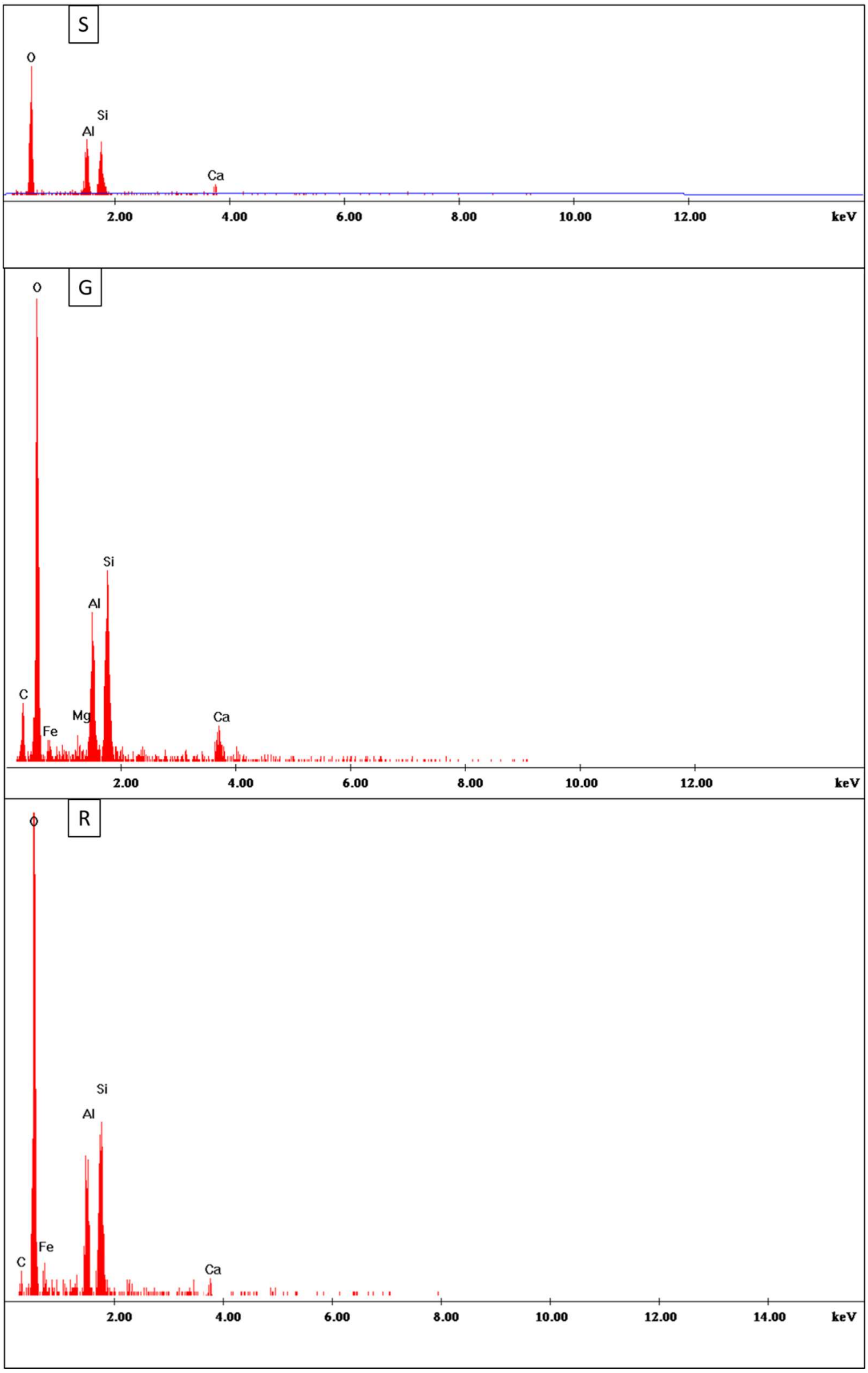

Figure S2. Spectrographs generated in the EDX analysis of Sopradeira cave samples. One spectrogram of sediment (S), gravel-like (G) and rock (R) samples.

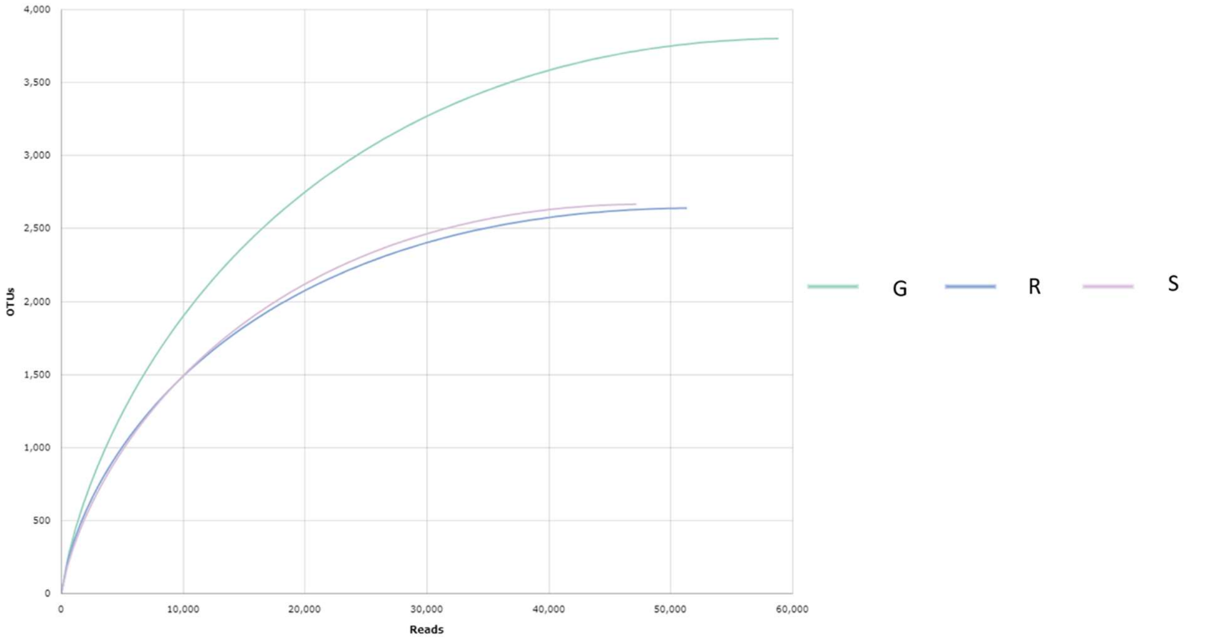

Figure S3. Rarefaction curves of rock, sediment and gravel-like samples from Sopradeira cave

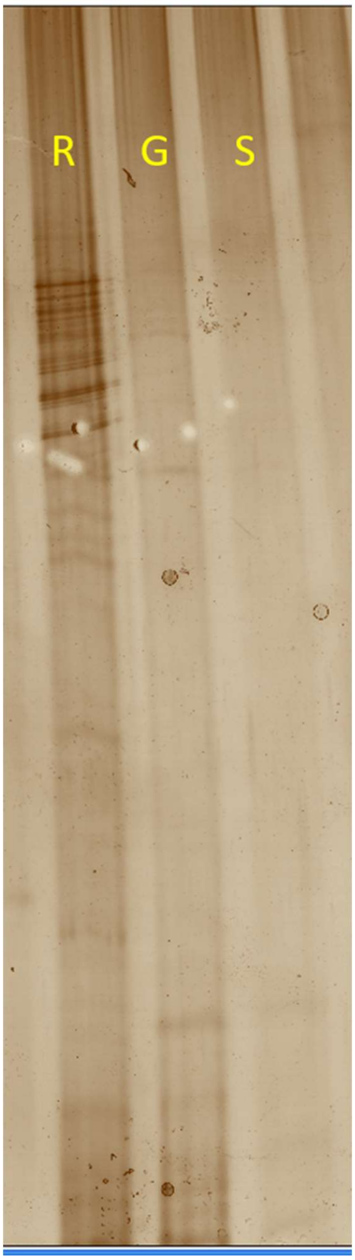

Figure S4. Denaturing Gradient Gel electrophoresis (DGGE) of *nifH* gene community in the rock (R), sediment (S) and gravel-like (G) samples. DGGE were made in an 8% polyacrylamide gel in a denaturing gradient of 35-65% following Marques et al., 2018 [4].
